# Effect of *p*-Synephrine on Fat Oxidation Rate during Exercise of Increasing Intensity in Healthy Active Women

**DOI:** 10.3390/nu14204352

**Published:** 2022-10-17

**Authors:** Jorge Gutiérrez-Hellín, Millán Aguilar-Navarro, Carlos Ruiz-Moreno, Alejandro Muñoz, Francisco J. Amaro-Gahete, María Posada-Ayala, Álvaro López-Samanes, Juan Del Coso, David Varillas-Delgado

**Affiliations:** 1 Exercise and Sport Science, Faculty of Health Sciences, Universidad Francisco de Vitoria, 28223 Pozuelo, Spain; 2 Exercise Physiology Laboratory, Camilo José Cela University, 28692 Villanueva de la Cañada, Spain; 3 Department of Medical Physiology, School of Medicine, University of Granada, 18071 Granada, Spain; 4 Faculty of Experimental Sciences, Universidad Francisco de Vitoria, 28223 Pozuelo, Spain; 5 School of Physiotherapy, Faculty of Health Sciences, Francisco de Vitoria University, 28223 Madrid, Spain; 6 Centre for Sport Studies, Rey Juan Carlos University, 28943 Fuenlabrada, Spain

**Keywords:** sports nutrition, phytochemical, weight loss, endurance exercise, women athletes

## Abstract

*p*-Synephrine is the principal alkaloid of bitter orange (*Citrus aurantium*). Several recent investigations have found that the intake of 2–3 mg/kg of *p*-synephrine raises fat oxidation rate during exercise of low-to-moderate intensity. However, these investigations have been carried out only with samples of male participants or mixed men/women samples. Therefore, the aim of this investigation was to study the effect of *p*-synephrine intake on fat oxidation during exercise of increasing intensity in healthy women. Using a double-blind, randomized experiment, 18 healthy recreationally active women performed two identical exercise trials after the ingestion of (a) 3 mg/kg of *p*-synephrine and (b) 3 mg/kg of a placebo (cellulose). The exercise trials consisted of a ramp test (from 30 to 80% of maximal oxygen uptake; VO_2_max) on a cycle ergometer while substrate oxidation rates were measured at each workload by indirect calorimetry. In comparison to the placebo, the intake of *p*-synephrine increased resting tympanic temperature (36.1 ± 0.5 vs. 36.4 ± 0.4 °C *p* = 0.033, *d* = 0.87) with no effect on resting heart rate (*p* = 0.111) and systolic (*p* = 0.994) and diastolic blood pressure (*p* = 0.751). During exercise, there was no significant effect of *p*-synephrine on fat oxidation rate (F = 0.517; *p* = 0.484), carbohydrate oxidation rate (F = 0.730; *p* = 0.795), energy expenditure rate (F = 0.480; *p* = 0.833), heart rate (F = 4.269; *p* = 0.068) and participant’s perceived exertion (F = 0.337; *p* = 0.580). The maximal rate of fat oxidation with placebo was 0.26 ± 0.10 g/min and it was similar with *p*-synephrine (0.28 ± 0.08 g/min, *p* = 0.449, *d* = 0.21). An acute intake of 3 mg/kg of *p*-synephrine before exercise did not modify energy expenditure and substrate oxidation during submaximal aerobic exercise in healthy active women. It is likely that the increase in resting tympanic temperature induced by *p*-synephrine hindered the effect of this substance on fat utilization during exercise in healthy active women.

## 1. Introduction

The alkaloid *p*-synephrine (4-[1-hydroxy-2-(methylamino)ethyl]phenol), is naturally present in the bitter orange (*Citrus aurantium*) and other fruits of the *Rutaceae* family [1]. As the primary phytochemical of the bitter orange, *p*-synephrine represents 0.10–0.35% of the weight of the fruit in the natural form, although it can reach ~3.00% in dry extracts [2]. The concentration of *p*-synephrine in some commercially available dietary supplements can reach up to 19% of product weight through extraction of products that naturally contain *p*-synephrine [3]. The manufacturing process of bitter orange extracts allows the consumption of considerable amounts of *p*-synephrine through dietary supplements without the need of consuming large quantities of the bitter orange fruit. Additionally, the *p*-synephrine can be chemically isolated from bitter orange and other citrus fruits, and there are products on the market that contain *p*-synephrine (pure) as their only ingredient. The use of *p*-synephrine gained popularity in the market of weight-loss supplements when Ephedra species were banned by the Food and Drug Administration of the United States in 2004 [4], due to the structural similarity of *p*-synephrine and ephedrine. In that context, citrus aurantium, and, consequently, *p*-synephrine, was habitually included in weight-loss products instead of ephedrine because of its purportedly thermogenic effects at rest [5,6,7]. However, even today, the literature confirming the potential benefits of *p*-synephrine to enhance weight loss, or facilitate body composition changes, is very limited and contradictory.

Recently, a few investigations have been carried out to evaluate the effect of *p*-synephrine on fat oxidation during exercise. In these investigations, the acute intake of 2–3 mg/kg of pure *p*-synephrine was effective in enhancing the use of fat as a fuel during exercise of submaximal intensity, without modifying pre-exercise blood pressure, exercise heart rate, energy expenditure rate and perceived exertion [8,9,10,11,12]. In most of these investigations, the effect of *p*-synephrine on fat oxidation was established by using an adapted version of the “Fatmax” test [8,9,10,12]. The Fatmax test was originally proposed by Achten et al. [13] and consists of exercise (habitually on a cyclergometer) of increasing intensity (at least until the respiratory quotient is >1.0), while fat oxidation is measured at each intensity by indirect calorimetry. The Fatmax test allows the elucidation of whole-body fat oxidation rates across a range of exercise intensities within the same experimental session. Additionally, it permits assessment of the maximal rate of fat oxidation (MFO) during exercise, and the intensity at which the MFO occurs (i.e., Fatmax [14]). The curve that explains the relationship between fat oxidation rate and exercise intensity has a negative parabolic shape and MFO is normally obtained when exercising at moderate intensity (between 40 to 60% of VO2max, depending on the aerobic fitness level of the individual). In previous investigations [8,9,10,12], the acute intake of *p*-synephrine, 45–60 min before exercise, produced a fat oxidation rate–exercise intensity curve that was displaced upwards, suggesting an effect of this phytochemical in enhancing fat utilization within a wide range of submaximal exercise intensities [8,9,10,12], including an enhancement in MFO. Additionally, *p*-synephrine intake increased MFO without modifying Fatmax, indicating that this substance can be used to enhance fat oxidation without the need to modify exercise intensity in training routines.

The increase in fat oxidation rate with *p*-synephrine in previous investigations was accompanied by a concomitant reduction in carbohydrate oxidation rates, producing the above-mentioned neutral effect of this substance on energy expenditure during exercise [8,9,10,11,12]. The higher fat oxidation rates at moderate exercise intensities with *p*-synephrine, at the expense of lower carbohydrate use, might help to spare muscle and liver glycogen during exercise. However, the usefulness of the *p*-synephrine-induced reduction on carbohydrate oxidation during exercise has not been properly investigated and requires further experimentation. These previous investigations have also reported minimal side effects of *p*-synephrine in the 24-h period after ingestion, when compared to the ingestion of a placebo [8,9,10,11,12]. This has constituted evidence to support *p*-synephrine being considered a safe substance, at least for young and healthy individuals [15].

The effect of *p*-synephrine on fat oxidation during exercise has been found in active individuals with different profiles [9,10,12] and in highly trained athletes [11]. In these investigations, *p*-synephrine increased fat oxidation by ~0.10–0.20 g/min, which renders a few extra grams of fat being oxidized per hour of exercise. For this reason, the general recommendations about *p*-synephrine use in the context of exercise programs for weight loss/body composition change suggest that this substance must be ingested in the long-term to produce measurable effects on weight loss/body composition.

Interestingly, all the aforementioned investigations have been carried out in samples of male participants or mixed-gender samples where women were only a small portion of the study sample (only 5 of the 64 participants included in previous investigations were women: 7.8% of the total sample). Hence, it is unknown whether acute *p*-synephrine intake may have the same impacts on fat oxidation during exercise in women as reported in men [16]. Therefore, the aim of this study was to evaluate the effect of the acute ingestion of 3 mg/kg of *p*-synephrine on the rate of fat oxidation during exercise of increasing intensity in healthy active women.

## 2. Materials and Methods

### 2.1. Participants

Eighteen healthy and recreationally active women volunteered to participate in this study. Participants’ characteristics are depicted in Table 1. The inclusion criteria for potential participants were: (a) regular aerobic exercise training of ≥1 h per day, ≥4 days per week for the previous year and (b) age between 18 and 45 years, (c) regular menstrual cycles measured for the prior 4 months before testing. Exclusion criteria were: (a) smoking status, (b) using medications -including oral contraceptive pills- or any type of dietary supplements in the month before testing, (c) cardiopulmonary or musculoskeletal disease, (d) presence of any menstrual disorder. Before the onset of the study, participants were notified of the experimental procedures and potential hazards associated with the experiment. Once inclusion/exclusion criteria were applied, all participants signed a written informed consent form to participate in the research. The study was approved by the Ethics Committee of the Universidad Francisco de Vitoria (UFV 18/2020) and was conducted in accordance with the latest version of the Declaration of Helsinki.

### 2.2. Sample Size Calculation

An a priori sample size calculation was performed by using the G*Power software (v.3.1.9.7, Germany) and data from a previous investigation on the effect of 3 mg/kg of *p*-synephrine on fat oxidation rate in male cyclists [8]. The sample size was calculated to obtain an effect size of 0.45 Cohen’s d units with a statistical power of 0.95, a two-tailed α level of 0.05, for an analysis of variance (ANOVA) of repeated measures, within factors (one group, 6 measurements) and a non-sphericity correction of 0.6. The sample size calculation showed that ≥14 individuals were needed to obtain statistically significant differences between *p*-synephrine and placebo on the maximal rate of fat oxidation during exercise. We recruited 18 women to increase the likelihood of finding differences between the treatments as no previous study used women as a study sample.

### 2.3. Experimental Procedure

A randomized, counterbalanced, double-blind and placebo-controlled experimental design was used for this study. Each woman completed a pre-experimental trial, a familiarization trial, and two identical experimental trials. All trials were separated by at least 3 days to allow for a full recovery and substance washout. In the two experimental trials, participants ingested (a) 3 mg of *p*-synephrine (99.2% purity; Synephrine HCL, LIFTMODE, USA) per kg of body mass (absolute dose = 185 ± 29 mg), (b) the same amount of a placebo (cellulose; 100% purity, Guinama, La Pobla de Vallbona, Spain). The substances were administered in an opaque, unidentifiable capsule and ingested with 150 mL of water 60 min before the beginning of the exercise test. A code was assigned to all trials and the order of trials was randomized by an independent investigator to allow substance identification by participants and experimenters. The regularity and length of the menstrual cycle of each participant were monitored for 4 months before the start of the study using a mobile app (Mycalendar, Period-tracker, USA). Participants completed a menstrual diary with this app that included: the duration of menstruation, dates of menstruation and level of discomfort during the days before the menstrual activity. These controls were obtained to ensure that each participant performed both experimental tests in the same phase either follicular or luteal- of her menstrual cycle. Laboratory temperature and humidity were registered and controlled (21.7 ± 0.8 °C and 42.5 ± 5.3%, respectively) to minimize the effect of these variables on substrate oxidation during exercise [17]. All experimental trials were carried out in the morning to prevent the bias of circadian rhythm on the variables under investigation [18].

### 2.4. Pre-Experimental and Familiarization Trials

One week before the onset of the study, participants were weighed and body height was obtained by a scale/stadiometer (model 700, Seca, Hammer Steindamm, Germany). Then, the body composition of each participant was estimated by bioelectrical impedance analysis (InnerScan Dual, Tanita, Tokyo, Japan). Afterwards, the participants underwent a ramp exercise test on a cycle ergometer (Ergoselect 4, Ergoline, Bitz, Germany) until voluntary fatigue to assess their VO2max. During the test, the workload was increased by 15 W/min and oxygen consumption (VO2) and carbon dioxide production (VCO2) were continuously measured with a breath-by-breath analyzer (Ergostik, Geratherm Respiratory, Bad Kissingen, Germany). VO2max was defined as the highest VO2 value obtained during the test. Habitual VO2max criteria were set to ensure that the test was maximal [19]. The data obtained in this pre-experimental test were used to normalize exercise intensity in the experimental trials as a percentage of individual VO2max values. Within the week prior to the first experimental trial, a second pre-experimental trial was carried out to familiarize participants with the procedures described below.

### 2.5. Experimental Trials

Twenty-four hours before each experimental trial, participants were advised to avoid vigorous exercise and they adopted a pre-competition-like diet and fluid intake regime. Participants were also required to avoid *p*-synephrine, orange juice, citrus-fruit derivatives, alcohol, caffeine, and other stimulants in the 24-h before each test. The fulfilment of these standardizations was confirmed with diet and training diaries. Participants were also asked to complete a 24-h dietary recall the day before the first trial and to adhere to the same dietary pattern 24 h before the second trial. In each trial, participants arrived at the laboratory between 08:00 and 10:00 AM, in a fasted state (at least 8 h after their last meal) and 2 h after ingesting 7 mL/kg of water to increase the likelihood of euhydration. Upon arrival, participants voided and euhydration was confirmed by urine specific gravity < 1.020 [20]. Before each trial, a bottle with accredited calibration gases (16.0% O_2_; 5.0% CO_2_, Sanro, Madrid, Spain) was used to calibrate the gas analyzer and a certified syringe of 3 L was used to calibrate the flowmeter.

Once all the standardizations were satisfied, the participant ingested the capsule assigned for the trial, and this ingestion was verified by a researcher, and then rested supine for 60 min. In the last 5 min of this period, a record of systolic and diastolic blood pressure (M6 Comfort, Omron, Kyoto, Japan) and heart rate (Wearlink+V800, Polar, Kempele, Finland) were obtained. At this point, the tympanic temperature was measured by triplicate after removal of earwax, when needed, in the left auditive canal by using an infrared tympanic thermometer (Thermoscan 7 IRT6520, Braun, Melsungen, Germany). Thereafter, participants voided, and a urine sample was obtained to measure pre-exercise urine concentrations of *p*-synephrine and 4-hydroxymandelic acid. After these resting measurements, participants wore cycling clothes to start the ramp exercise test.

Warm-up consisted of 10 min at 30% of their VO_2_max on the cycle ergometer. Then, participants started the exercise trial at 30% of VO_2_max for 3 min followed by 10% increments in VO_2_max every 3 min until they reached the workload equivalent to 80% of VO_2_max. At the end of each 3-min stages, the rating of perceived exertion was recorded by using the traditional Borg scale [21]. During exercise, expired gases were collected and measured and representative values of VO_2_ and VCO_2_ and heart rate were recorded by averaging values measured for the last 60 s of each 3-min stage [22]. Energy expenditure and substrate oxidation rates were calculated from stoichiometric equations [23,24]. In each trial, MFO was individually calculated for each participant (using the last 60 s of each stage). Fatmax was established as the % of VO_2_max at which MFO occurred.

Once participants finished the exercise protocol, they continued with their daily activities but were encouraged to avoid exercise and the intake of stimulants, as in the 24-h prior to the trial. On the following morning, participants completed an ad hoc questionnaire regarding side effects derived from stimulant intake [25]. This questionnaire included a 1-to-10 arbitrary units (a.u.) scale to assess the magnitude of nervousness, vigor, irritability, gastrointestinal problems, muscular pain, headache, diuresis, and insomnia. The questionnaire was filled out ~24 h after *p*-synephrine or placebo intake.

### 2.6. Urine p-Synephrine and 4-hydroxymandelic Acid Concentrations

The urine samples were frozen at −20 °C and measured afterwards. On a later date (~1 month after collection), *p*-synephrine and 4-hydroxymandelic acid extraction from urine samples was carried out following procedure: pH was adjusted to 3 with the addition of formic acid (Merck, Darmstadt, Germany) in a concentration of 0.1%. A volume of 1 mL was centrifugated for 10 min at 4000 rpm and 4 °C. Afterwards, the aliquot sample was analyzed on a QTrap 4500 mass spectrometry system (Sciex, Darmstadt, Germany) equipped with a Turbo V electrospray ionization source. Mass spectrometry was coupled with an Acquity UPLC H-Class (Waters, Mildford, CT, USA). The chromatography was performed at 30 °C with a Avantor ACE (VWR, Reading, England) C18-PFP column (100 × 2.1 mm, i.d. 1.7 µm) using a mobile phase composed of formic acid 0.1% (A) and methanol (B). A gradient elution at a flow rate 300 µL/min was applied: 0–2 min, 10%B; 2–3 min, 10–20%B; 3–4 min, 20%B; 4–4.5 min, 20–50%B; 4.5–5 min, 50–80%B; 5–5.1 min, 80–20%B; 5.1–6 min, 20–0%B; 6–7min, 0%B; 7–7.1 min, 10%B, 7.1–10 min, 10%B; and returned to initial conditions. The injection volume of the samples was 5 µL. Calibration curves were constructed using commercially available standards, (99.2% purity; *p*-synephrine HCL, LIFTMODE, USA; 4-hydroxymandelic acid, ThermoFisher, Waltham, MA, USA), in the range 0.05–60 µg/mL. All urine samples were analyzed by triplicate intraday. The limit of detection and limit of quantification were used to determine the linearity. Calibration range, carryover, accuracy, and precision (within-run and between-run), selectivity, recovery and matrix effect were evaluated.

### 2.7. Statistical Analysis

The results of each test were entered in a blinded fashion into the SPSS v21.0 statistical package and analyzed afterwards by organizing data from trials with ingestion of *p*-synephrine vs. placebo. Before the onset of the comparison of *p*-synephrine vs. placebo, the normality of each variable was checked with the Shapiro-Wilk test. All variables were normally distributed, and parametric statistics were applied. *T*-tests for paired samples were employed to compare cardiovascular variables and tympanic temperature at rest, MFO, Fatmax, urine *p*-synephrine and 4-hydroxymandelic acid concentrations and the magnitude of side effects derived from *p*-synephrine vs. placebo intake. A two-way ANOVA (substance × exercise intensity; 2 × 6) was used to compare the effect of *p*-synephrine on energy expenditure, fat, and carbohydrate oxidation rates, heart rate and the rating of perceived exertion during exercise. For these variables, the ANOVA’s main effects of substance and exercise intensity, in addition to the interaction between these two effects, were calculated. In the case of an F significant test, the LSD post hoc test was applied to detect differences in the pairwise comparisons between *p*-synephrine and placebo at the same intensity. The Cohen’s effect size (*d*) was calculated in all pairwise comparisons between *p*-synephrine and placebo and they were interpreted according to the following thresholds: <0.20 trivial, ≥0.20–0.59 small, ≥0.60–1.19 moderate, ≥1.20–1.99 large, and ≥2.00 very large [26]. The data are presented as mean ± standard deviation (SD). The level of significance was set at *p* < 0.050. The potential existence of an order effect was tested by comparing data on first vs. second experimental trial with the same statistical methods described for the *p*-synephrine vs. placebo comparison. There were no differences between the first and second experimental trial in any of the variables under investigation.

## 3. Results

In comparison to the placebo, the acute ingestion of *p*-synephrine did not modify resting heart rate (*p* = 0.111), and systolic blood (*p* = 0.994) and diastolic blood pressure (*p* = 0.752, Table 2). However, *p*-synephrine intake increased tympanic temperature at rest (*p* = 0.033).

**Figure 1:**
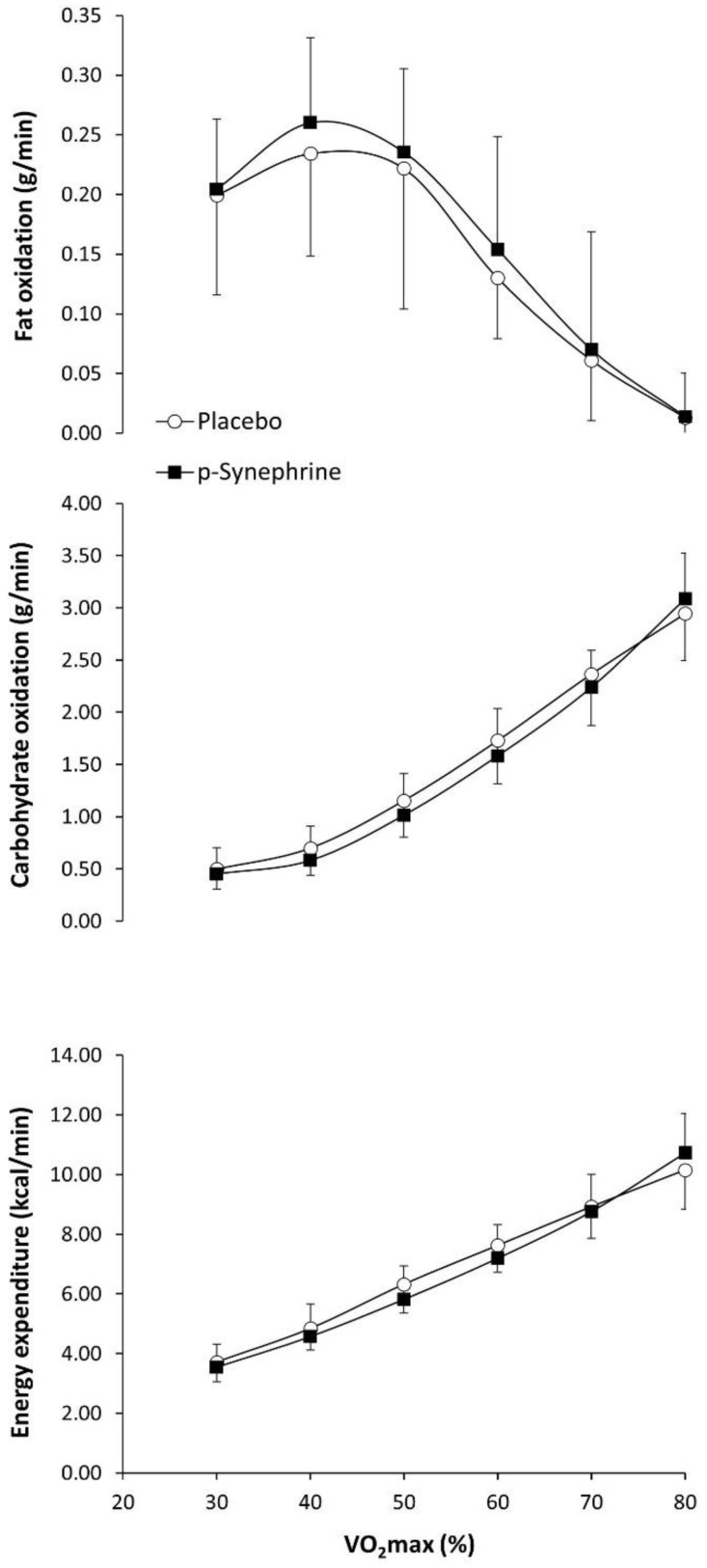
Fat oxidation, carbohydrate oxidation and energy expenditure during exercise of increasing intensity after the ingestion of 3 mg/kg of *p*-synephrine or a placebo in healthy active women.

Figure 1 depicts the effect of *p*-synephrine on the rates of fat oxidation, carbohydrate oxidation, and energy expenditure during exercise of increasing intensity. There was a significant effect of exercise intensity on fat oxidation rate (F = 35.704; *p* < 0.001) with no effect of substance (F = 0.517; *p* = 0.484) or substance × exercise intensity interaction (F = 0.856; *p* = 0.542). The magnitude of *d* was between 0.01 and 0.28 for the effect of *p*-synephrine on fat oxidation over the placebo during exercise. There was a significant effect of exercise intensity on carbohydrate oxidation rate (F = 32.974; *p* = 0.030) with no effect of substance (F = 0.730; *p* = 0.795) or substance × exercise intensity interaction (F = 1.967; *p* = 0.370). The magnitude of *d* was between −0.40 and 0.31 for the effect of *p*-synephrine on carbohydrate oxidation over the placebo. There was a significant effect of exercise intensity on energy expenditure rate (F = 53.553; *p* = 0.018) with no effect of substance (F = 0.480; *p* = 0.833) or substance × exercise intensity interaction (F = 0.481; *p* = 0.780).

**Figure 2:**
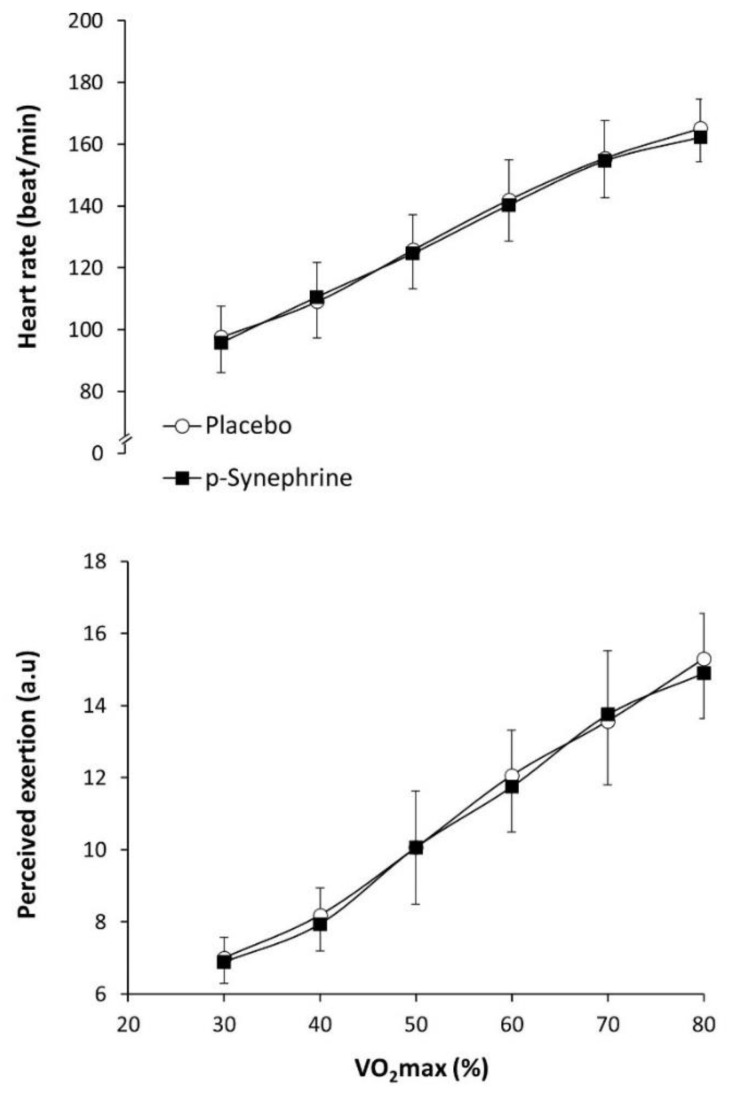
Heart rate and rating of perceived exertion during exercise of increasing intensity after the ingestion of 3 mg/kg of *p*-synephrine or a placebo in healthy active women.

Figure 2 depicts the effect of *p*-synephrine intake on heart rate and the rating of perceived exercise and during exercise. There was a significant effect of exercise intensity (F = 44.746; *p* = 0.005) on heart rate, while no effect of substance (F = 4.629; *p* = 0.068) or substance × exercise intensity interaction was reported for this variable. There was also a significant effect of exercise intensity (F = 30.234; *p* = 0.009) on the rating of perceived exertion. However, there was no effect of substance on the rating of perceived exertion (F = 0.337; *p* = 0.580) and the substance × exercise intensity interaction did not reach statistical significance.

**Figure 3:**
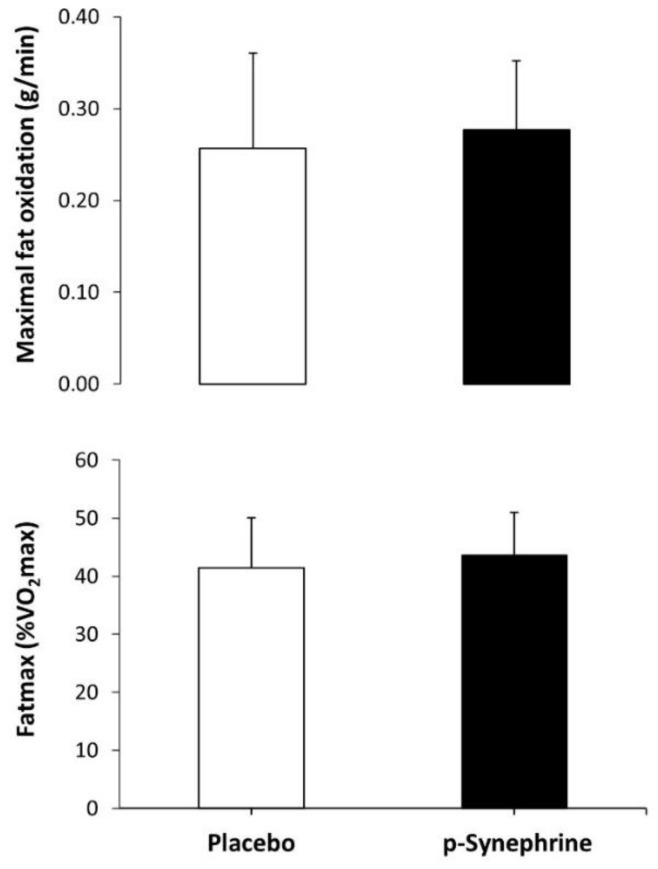
Maximal fat oxidation rate and exercise intensity at maximal fat oxidation (Fatmax) during exercise of increasing intensity after the ingestion of 3 mg/kg of *p*-synephrine or a placebo in in healthy active women.

Figure 3 shows the effect of *p*-synephrine on MFO and Fatmax. The intake of *p*-synephrine did not modify MFO (0.28 ± 0.08 vs. 0.26 ± 0.10 g/min, *p* = 0.449, *d* = 0.21, respectively) or Fatmax (44.6 ± 7.8 vs. 40.8 ± 8.6%, *p* = 0.096, *d* = 0.50, respectively) over the placebo condition.

Within 24 h of *p*-synephrine ingestion, participants reported higher ratings of irritability and gastrointestinal distress in comparison to the placebo (Table 3; *p* < 0.050). There were no other statistically significant differences in the magnitude of the remaining side effects between *p*-synephrine and placebo ingestion.

The intake of *p*-synephrine increased urinary concentrations of both *p*-synephrine (*p* = 0.006) and 4-hydroxymandelic acid concentrations over the placebo (*p* = 0.028) (Table 4).

## 4. Discussion

In this double-blind, placebo-controlled study involving healthy active women, a single dose of 3 mg of *p*-synephrine per kg of body mass did not significantly increase the rate of fat oxidation during aerobic exercise of submaximal intensity The current investigation is innovative because it is the first to examine the effect of *p*-synephrine intake on substrate oxidation during exercise in women but contradicts the findings of several previous investigations [8,9,10,11,12]. The reason(s) for the lack of agreement between the current experiment and previous investigations is(are) not evident, as the type and dose of *p*-synephrine administered to the participants, the time of ingestion prior to exercise, the ramp exercise test employed and the methods to measure fat oxidation are almost identical among investigations [8,9,10,12]. The only appreciable difference among investigations is the sex of the sample: only women participated in the current study while previous investigations recruited only men or used samples of mixed gender where women were only a minor proportion [8,9,10,12]. The comparison among investigations points towards a sex-specific difference in the response to acute *p*-synephrine intake. Although future investigations are needed to confirm the main result of this study, it seems that women seeking enhanced fat oxidation during exercise may not benefit from the intake of 3 mg/kg of *p*-synephrine.

A recent systematic review summarizing the findings of studies published on this topic [16] has concluded that acute *p*-synephrine intake increases the reliance on fat during low- to moderate-intensity exercise (from 30–80% of VO2max), and this effect is present in participants with different aerobic fitness levels. This benefit may be obtained when *p*-synephrine is ingested in doses between 2–3 mg per kg of body mass, ingested about 60 min before exercise, and during exercise forms that include steady-state exercise and ramp exercise protocols. The enhancement in fat utilization with *p*-synephrine is normally modest, as it is habitually associated with an improvement of ~0.1 g of fat oxidized per min of exercise. The effect of *p*-synephrine on fat oxidation is obtained without any change in Fatmax, likely due to the lack of impact of this substance on exercise performance [27]. There is data suggesting that the effect of *p*-synephrine to increase the rate of fat oxidation may be maintained after exercise [28], although this effect requires further research as it has been reported in only one investigation. It has been reported that *p*-Synephrine also reduces the rate of carbohydrate oxidation during exercise of low to moderate intensity while energy expenditure, heart rate and participants’ feelings of fatigue are generally unchanged. In the view of the previous literature, *p*-synephrine is considered effective to enhance fat oxidation and safe. However, the outcomes of the current experiment add some information to the literature on this topic, as this phytochemical was not effective in modifying substrate during exercise in women. Although the effect of *p*-synephrine on MFO was small in magnitude (*d* = 0.21), and the curve of fat oxidation rate-exercise intensity was displaced upwards, as in previous investigations (see Figure 1 and [8,9,10,11,12]), the main effect of *p*-synephrine was far from reaching statistical significance. Collectively, all this information suggests that all the aforementioned benefits of *p*-synephrine intake during exercise may be specific (or at least amplified) for men.

In the current experiment, we obtained data on heart rate and blood pressure at rest and the ingestion of *p*-synephrine did not produce any modification 60 min after ingestion when compared to the ingestion of the placebo (Table 2). This is a recurrent finding in previous investigations [6] and supports the innocuous nature of this substance when ingested at doses up 3 mg/kg, as its effect on cardiovascular variables is negligible. Sixty min after substance ingestion, we also measured urine concentrations of *p*-synephrine and 4-hydroxymandelic acid to ensure that the substance was adequately absorbed and metabolized in our sample of healthy active women. Metabolism of *p*-Synephrine occurs in the liver, where it is broken down into different metabolites, the most important of which is 4-hydroxymandelic acid, which is excreted in the urine [29]. Interestingly, the values of urinary *p*-synephrine were half the value of the ones found in urine samples of elite sprinters (11 men and 2 women) [27], while the concentration of 4-hydroxymandelic acid was 30 times lower in our sample of women participants, with respect to the values found in mixed-gender sprinters [27]. Although in both studies the dose of *p*-synephrine used was equal (3 mg/kg), the comparison of the results of urinary concentrations between studies should be made with care as the lapse between ingestion and urine collection was substantially different (60 min in the current study vs. ~180 min in the sprinters’ study; [27]). Nevertheless, the great differences found in the urine concentration of 4-hydroxymandelic acid between studies may reveal some sex-specific differences towards a lower rate of *p*-synephrine metabolism and excretion in women that may require further experimentation.

In addition to cardiovascular variables and urine samples, we obtained data on tympanic temperature at rest. Although this measurement was initially included as a control, participants presented +0.3 °C of tympanic temperature after the ingestion of *p*-synephrine than after the ingestion of the placebo (Table 3), representing an effect of moderate magnitude. This effect of *p*-synephrine on resting tympanic temperature was present despite the fact that we carefully placed the two experimental trials for each participant within the same phase of the menstrual cycle. Specifically, 4 participants (23.3% of the sample) performed the placebo and *p*-synephrine trials between the 5th and 9th days of their menstrual cycle, just after the end of menstruation and before ovulation. The remaining 14 participants (77.7%) performed the placebo and *p*-synephrine trials between the 18th and 22nd days of their menstrual cycle, just in the middle of their luteal phase. Hence, although it is well known that basal body temperature increases by ~0.3 °C with ovulation and remains raised during the whole luteal phase with respect to the follicular phase [30], the fluctuations on basal body temperature due to the menstrual cycle were not responsible for the increase found in the current investigation. Thus, we assume that body temperature at rest increased due to the ingestion of *p*-synephrine. A previous study found that, after the acute ingestion of a dietary supplement containing *p*-synephrine, caffeine, and guarana, among other ingredients, women presented an increase in skin temperature that was not present in men [31]. Interestingly, these effects in skin temperature in women were present despite heart rate and blood pressure having changed equally in men and women during the 6-h period of measurement of this investigation. Additionally, there are data to confirm that women’s responses to Citrus aurantium are of lower magnitude than those of men, with lower increases in energy expenditure and a diminished effect on reducing the respiratory quotient at rest [32]. Lastly, although there is no evidence in humans, a study comparing body weight evolution of male and female rats exposed to solutions containing *p*-synephrine, ephedrine, salicin, and caffeine (proportions of 10:4:6:80, respectively) for 28 days found that females were less susceptible to the effects of the *p*-synephrine-containing product on body weight fluctuations [33]. Collectively, all this information supports a potential sex-derived effect on the physiological outcomes derived from the intake of *p*-synephrine-containing products. Briefly, while this substance may be effective to enhance fat oxidation during exercise in men, the efficacy of *p*-synephrine to produce a shift in substrate oxidation in women may be dampened by a “thermogenic” effect of this substance that produces an increase in body temperature. As a small increase of ~0.3–0.5 °C in tympanic temperature already reduces the utilization of fat during exercise [17], we suggest that the higher resting tympanic temperature in the *p*-synephrine trial dampened the effect of this substance on fat oxidation in women. At this moment, this is a hypothesis that should be verified with further investigation using studies that include a direct comparison of acute responses to *p*-synephrine intake in men and women.

In healthy humans, the acute intake of pure *p*-synephrine has not been linked to severe drawbacks, while the side effects reported in some *p*-synephrine-containing products are habitually linked to other stimulants included in dietary supplements (e.g., caffeine or ephedrine [6,16]). In animal models, the safety of *p*-synephrine is certified up to doses of 1000 mg/kg/day [34]. In the current investigation, women reported higher irritability and gastrointestinal distress with *p*-synephrine than with the placebo. Again, this is a novel finding because previous investigations in men or mixed-gender samples did not report such side effects [8,9,10,11,12,27], despite the same dosage and form of administration of *p*-synephrine. Although these side effects may be another proof of the different responses to *p*-synephrine in women vs. men, the magnitude of the drawbacks (participants rated them as ~2 a.u., on a 1–10 a.u. scale) suggests that *p*-synephrine can be considered as a safe substance, at least for healthy individuals.

Despite the strengths and novelty of this investigation, the study has some limitations: (i) the present investigation was only conducted with healthy active women, and, therefore, no extrapolation should be assumed for other populations, such as men, women athletes, or clinical populations. As indicated above, the same dose of *p*-synephrine produced an increase in fat oxidation during exercise in samples of men or mixed-gender samples. It is well established that women rely more on fat and less on carbohydrates at the same relative exercise intensity than men [35], likely because sex steroids play a pivotal role in the sex-related differences in substrate oxidation during exercise. Further investigations in women, with measurements of the potential effect of *p*-synephrine on fat oxidation during exercise should be accompanied with assessments of blood progesterone and oestradiol concentrations. These types of studies will be key to determine if there is a potential interference of female sex hormones on the effect of *p*-synephrine; (ii) only a 3 mg/kg dose of *p*-synephrine was examined and no association with substrate oxidation was found. From the current data, we cannot conclude that *p*-synephrine is ineffective to enhance fat oxidation during exercise, as higher doses may provide greater benefits; (iii) no data on the potential mechanism of action of *p*-synephrine to enhance fat oxidation was obtained; therefore, we cannot determine why 3 mg/kg *p*-synephrine were not effective in shifting substrate oxidation during exercise. Future investigation should be carried out to determine the changes in free fatty acid concentration with different doses of *p*-synephrine in healthy active female subjects and the measurement of female sex hormones to examine the possible interaction between them and *p*-synephrine metabolism; (iv) we used an exercise protocol of increasing intensity that helps to identify the maximal rate of fat oxidation during submaximal aerobic exercise (i.e., the Fatmax test). However, this type of exercise is not a typical training routine for exercise practitioners who seek body mass loss or body composition changes. Thus, the effects of *p*-synephrine in women should be confirmed by using prolonged and constant-load exercise protocols, or by using more ecological exercise training situations.

## 5. Conclusions

An acute intake of 3 mg/kg of *p*-synephrine before exercise did not modify substrate oxidation during submaximal aerobic exercise in healthy active women. This outcome is contradictory to previous literature on male participants and mixed samples of men/women where a similar dose of *p*-synephrine increased fat oxidation rate during aerobic exercise of low-to-moderate intensity. It is likely that the *p*-synephrine-induced effect on increasing resting tympanic temperature hindered the effect of this substance in women. Additionally, *p*-synephrine induced some side effects for the 24 h after ingestion which adds to the hypothesis that the physiological responses to this substance may have some sex-specific features.

## Figures and Tables

**Table 1:** Participants’ age, morphological characteristics, and values at the end of a VO_2_max test on a cycle ergometer. Data are presented as mean ± standard deviations (SD) with minimal and maximal values (range).

Variable (Units)	Mean ± SD	Range
Age (year)	26.9 ± 8.7	18.0–40.0
Body mass (kg)	62.3 ± 9.0	47.9–83.5
Body height (cm)	166.7 ± 6.7	162.0–175.0
Fat mass (%)	24.3 ± 5.5	15.0–39.2
VO_2_max (mL/kg/min)	39.7 ± 6.5	30.4–50.2
Maximal heart rate (beat/min)	179 ± 7	170–197
Maximal wattage in the VO_2_max test (W)	216 ± 37	170–300

**Table 2:** Cardiovascular variables and tympanic temperature at rest and urine specific gravity after the ingestion of 3 mg/kg of *p*-synephrine or a placebo. Data are presented as mean ± standard deviations (SD).

Variable (Units)	Placebo	*p*-Synephrine	*d*	*p* Value
Heart rate (beat/min)	54 ± 9	58 ± 12	1.12	0.111
Systolic blood pressure (mmHg)	108.8 ± 6.1	108.8 ± 6.6	0.00	0.994
Diastolic blood pressure (mmHg)	67.9 ± 5.2	68.5 ± 7.5	0.08	0.751
Tympanic temperature (°C)	36.1 ± 0.5	36.4 ± 0.4 *	0.87	0.033
Urine specific gravity	1.012 ± 0.061	1.010 ± 0.073	0.23	0.450

(*) Differences between placebo and 3 mg/kg of *p*-synephrine (*p* < 0.050).

**Table 3:** Frequencies of side effects during the 24 h following the ingestion of 3 mg/kg of *p*-synephrine or a placebo.

Variable (Units)	Placebo	*p*-Synephrine	*p* Value
Nervousness (a.u.)	1.2 ± 0.6	1.9 ± 1.8	0.236
Vigour (a.u.)	1.4 ± 0.7	2.1 ± 2.3	0.558
Irritability (a.u.)	1.3 ± 0.7	2.1 ± 1.3 *	0.043
Muscle pain (a.u.)	2.1 ± 1.7	2.2 ± 1.3	0.681
Headache (a.u.)	2.7 ± 3.0	3.9 ± 2.8	0.347
Gastrointestinal distress (a.u.)	1.2 ± 0.6	2.1 ± 1.8 *	0.044
Diuresis (a.u.)	1.8 ± 0.3	1.8 ± 1.1	0.829
Insomnia (a.u.)	2.9 ± 2.5	2.5 ± 2.6	0.574
Sleep quality (a.u)	7.2 ± 1.9	6.1 ± 2.4	0.092

(*) Differences between placebo and 3 mg/kg of *p*-synephrine (*p* < 0.050).

**Table 4:** Urine *p*-synephrine and 4-hydroxymandelic acid concentrations after the ingestion of 3 mg/kg of *p*-synephrine or a placebo.

Variable (Units)	Placebo	*p*-Synephrine	*d*	*p* Value
Urine *p*-synephrine concentration (µg/L)	84.21 ± 37.46	30,432.4 ± 4257.9 *	0.71	0.006
Urine 4-hydroxymandelic acid concentration (µg/L)	0.07 ± 0.05	2.96 ± 5.28 *	0.55	0.028

(*) Differences between placebo and 3 mg/kg of *p*-synephrine (*p* < 0.050).

## Data Availability

Not applicable.
